# Validity and reliability of the Finnish version of the Functioning Assessment Short Test (FAST) in bipolar disorder

**DOI:** 10.1186/s40345-015-0025-1

**Published:** 2015-05-01

**Authors:** Kirsi Suominen, Elina Salminen, Susanna Lähteenmäki, Tiina Tupala, Erkki Isometsä

**Affiliations:** City of Helsinki, Social services and health care, Department of mental health and substance abuse, Bipolar Disorder Research and Treatment Centre, Nordenskiöldinkatu 20, 00250 Helsinki, Finland; Department of Psychiatry, University of Helsinki and Helsinki University Hospital, Välskärinkatu 12, 00260 Helsinki, Finland

**Keywords:** Bipolar disorder, Functional assessment, FAST, Validity, Reliability, Rehabilitation, Psychology

## Abstract

**Background:**

The Functioning Assessment Short Test (FAST) was developed for the clinical evaluation of functional impairment of patients suffering from bipolar disorder. The aim of this study was to validate the Finnish version of FAST.

**Methods:**

Translation and back-translation of FAST were performed. Fifty patients with the Diagnostic and Statistical Manual of Mental Disorders-Fourth edition (DSM-IV) bipolar type I and II were interviewed at the Bipolar Disorder Research and Treatment Centre, City of Helsinki, Finland. Participants completed the FAST, the Social and Occupational Functioning Assessment Scale (SOFAS) of DSM-IV, and the Sheehan Disability Scale (SDS) as part of the assessment. Internal consistency and correlations between FAST and SOFAS and SDS were analysed. Twenty-five patients participated in a reliability assessment carried out 1 week apart by a different rater.

**Results and discussion:**

The internal consistency coefficient obtained was very good, with a Cronbach alpha of 0.870. Reliability of FAST was also found excellent (correlation between two measures *r* = 0.896, *p* < 0.001). A highly significant negative correlation between FAST and SOFAS scores was found (*r* = −0.723, *p* < 0.001). FAST and SDS were also highly significantly correlated (*r* = 0.742, *p* < 0.001).

**Conclusions:**

The psychometric validity and reliability of FAST in the Finnish sample of patients with bipolar disorder types I and II were good.

## Background

Bipolar disorder is frequently associated with functional impairment (Whiteford et al. 2013). However, the concept of functional impairment is complex, involving different domains of functioning and areas of life. Furthermore, measures assessing functional impairment in bipolar disorder have also varied widely across studies. Among global scales assessing functioning, the Global Assessment of Functioning scale (GAF) (First et al. 1997) and the Social and Occupational Functioning Assessment Scale (SOFAS) (Goldman et al. 1992) are the ones most commonly used. The Social Adjustment Scale (SAS) (Weissman and Bothwell 1976), the Short Form Health Survey (SF-36) (Ware et al. 1994), the RAND-36 (Hays and Morales 2001) and the WHO-DAS (Guilera et al. 2014) are also used frequently. Nevertheless, none of these scales was developed to assess specific areas of functional impairment in bipolar disorder. While there are advantages to using uniform measures across different mental disorders, such scales may lack sensitivity or precision in illness-specific impairments. For example, the distinction between hypomania and mania is defined by the severity of impairment due to symptoms of mania, which typically lead to specific types of impairment not commonly seen in other mental disorders. The QoL.BD scale (Michalak and Murray 2010) is a disorder-specific scale of quality-of-life for patients with bipolar disorder. However, although constructs of functioning and quality-of-life partly overlap, they are also distinct, with quality-of-life emphasizing more the subjective experience. Thus, there is a need for illness-specific measures of functioning to take into account the domains of disability relevant to the illness.

The Functioning Assessment Short Test (FAST) (Rosa et al. 2007) was developed for the clinical evaluation of functional impairment of patients suffering from mental disorders, particularly bipolar disorder. The psychometric properties of FAST in terms of reliability and validity have been found to be good in bipolar disorder (Rosa et al. 2007, Cacilhas et al. 2009, Aydemir and Uykur 2012, Moro et al. 2012, Barbato et al. 2013). FAST has good psychometric properties also in attention-deficit/hyperactivity disorder (Rotger et al. 2014) and in the first psychotic episodes (González-Ortega et al. 2010).

The aim of this study was to validate the Finnish version of FAST in the assessment of functional impairment in subjects with bipolar disorder.

## Methods

### Subjects

The study was conducted in the city of Helsinki, Department of Social Services and Healthcare, Bipolar Disorder Research and Treatment Centre. Fifty bipolar types I and II patients were recruited and evaluated in 1 September 2013 to 31 March 2014. The diagnosis of bipolar disorder was made using the Structured Clinical Interview for Diagnostic and Statistical Manual of Mental Disorders-Fourth edition (DSM-IV) Disorders (SCID-I/P) (First et al. 1997) and all available information, including psychiatric records and interviews with significant others. Subjects with psychotic symptoms and substance use dependence were excluded. The study protocol was approved by the ethics committee of HUCH. All subjects were informed about the study protocol, and written informed consent was obtained. Twenty-five voluntary subjects participated also in a reliability assessment 1 week later, conducted by a different interviewer.

### Variables

Information was gathered on socio-demographic, lifestyle, and clinical variables, including age, sex, marital status, education, employment status, information about sick leave and disability pension, smoking and previous psychoactive substance use, and the subject’s self-assessment of capability to work. The Social and Occupational Functioning Assessment Scale of DSM-IV (SOFAS) (Goldman et al. 1992) and the Sheehan Disability Scale (SDS) (Sheehan et al. 1996) were also applied.

### Functioning Assessment Short Test

FAST was developed by the Bipolar Disorder Program, Barcelona, Spain (Rosa et al. 2007). This instrument was designed to be used by a trained clinician. The time-frame investigated is the last 15 days before assessment. It comprises 24 items divided among the six specific areas of functioning. All of the items are rated on a four-point scale. The global score is obtained by summing the scores of each item; the higher the score, the more serious the difficulties. A more detailed description of the instrument is presented in the original paper (Rosa et al. 2007). FAST has been translated into English (Vieta 2010), Italian (Moro et al. 2012, Barbato et al. 2013), Portuguese (Cacilhas et al. 2009), and Turkish (Aydemir and Uykur 2012). FAST was translated into Finnish and then back-translated into English and approved by the researchers of the Bipolar Disorder Program, Barcelona (Rosa et al., 2007), who developed the scale. A researcher from the group trained the Finnish group to administer the FAST.

### Statistical analyses

Internal consistency was analysed using Cronbach’s alpha. Reliability between two independent researchers was examined using the intra-class correlation coefficient. Pearson’s correlation coefficient was used to investigate the correlation between FAST and SOFAS and SDS. Total scores of FAST in the three groups according to employment status and experienced work ability were compared using one-way ANOVA with the Tukey’s HSD test.

## Results

Fifty bipolar patients (27 BP I, 23 BP II) participated in the study (Table 1). Male subjects comprised 38% of the study population. The mean age of participants was 37.5 ± 12.1 years (range 20 to 70 years).

**Table 1:** **Socio-demographic and clinical characteristics of 50 bipolar patients**

	***N***	**%**
Gender		
Male	19	38
Female	31	62
Bipolar type I	27	54
Bipolar type II	23	46
Marital status		
Married or cohabiting	20	40
Not cohabiting	19	38
Divorced or widowed	11	22
Education		
University	13	26
College	13	26
Vocational school	9	18
No professional education	15	30
Employment status		
Employed	15	30
Unemployed	2	4
Sick leave	5	10
Disability pension	23	46
Others	5	10
Living		
Alone	21	42
With parents	1	2
With partner	25	50
Others	3	6
Smoking		
Never	10	20
Finished	13	26
Occasionally	7	14
Regularly	20	40
Psychoactive substance abuse		
Never	38	76
Occasionally	10	20
Regularly	2	4
	Mean	SD
Age (years)	37.5	12.1
FAST	24.5	11.3
SOFAS	65	12.9
Sheehan	14.5	7.7
Audit	5.9	5.7

All items of the FAST were answered by all patients. The internal consistency coefficient obtained for the total translated scale was good (Cronbach’s alpha = 0.870).

The reliability of the FAST, SOFAS, and Sheehan was measured by comparing the first and second evaluation scores using a correlation coefficient (Table 2). The correlation between total FAST scores for each subject at measurements 1 and 2 was excellent (correlation coefficient *r* = 0.896).

**Table 2:** **Test-retest reliability of the FAST, SOFAS, and SDS**

	**First evaluation** ***N*** **= 25**		**Second evaluation** ***N*** **= 25**		**Intraclass correlation**	***p***
	**Mean**	**SD**	**Mean**	**SD**		
FAST autonomy (items 1 to 4)	1.68	1.84	1.76	1.59	0.827	<0.001
FAST occupational functioning (items 5 to 9)	8.12	5.71	7.76	5.92	0.988	<0.001
FAST cognitive functioning (items 10 to 14)	3.68	2.58	4.40	2.60	0.787	<0.001
FAST financial issues (items 15 to 16)	1.04	1.14	0.56	0.71	0.563	0.02
FAST interpersonal relationships (items 17 to 22)	4.21	2.48	4.56	1.89	0.551	0.03
FAST leisure time (items 23 to 24)	1.52	1.61	1.32	1.22	0.557	0.03
FAST total	20.12	10.48	20.36	9.42	0.896	<0.001
SOFAS	65.68	11.34	64.72	11.79	0.793	<0.001
Sheehan	13.24	6.69	14.32	7.38	0.938	<0.001

Validity based on functional impairment according to clinician-rated SOFAS and self-rated SDS was also investigated. Patients with good functioning receive high scores in SOFAS and low scores in FAST. FAST and SOFAS scores correlated negatively at the first evaluation (*N* = 50, *r* = −0.723, *p* < 0.001) and at the second evaluation (*N* = 25) (*r* = −0.863, *p* < 0.001). Both FAST and SDS give low scores with good functioning. FAST and SDS were correlated at both first evaluation (*N* = 50, *r* = 0.742, *p* < 0.001) and second evaluation (*N* = 25, *r* = 0.773, *p* < 0.001). Validity based on functioning at work according to FAST and SDS domains was examined. FAST occupational functioning domain (8.86 ± 6.29) and SDS work domain (5.28 ± 3.15) correlated positively (*r* = 0.738, *p* < 0.001). FAST correlated positively also with employment status and self-experienced work ability (Table 3).

**Table 3:** **FAST, SOFAS, and SDS scores according to employment status and experienced work ability**

	**Scores**					
	Employment status					
	Employed or student *N* = 15	Unemployed *N* = 2	Sick leave or disability pension *N* = 28	Others *N* = 5	*F*	*p*
FAST	11.67 ± 5.65	17.50 ± 12.02	29.36 ± 8.20	11.00 ± 11.29	23.17	<0.001
SOFAS	74.93 ± 7.89	73.00 ± 7.07	57.54 ± 11.13	73.40 ± 8.96	19.85	<0.001
SDS	8.33 ± 4.77	6.00 ± 0.0	18.21 ± 6.83	12.80 ± 6.94	48.91	<0.001
	Experienced work ability					
	Good *N* = 10	Decreased *N* = 18	Unable to work *N* = 22		*F*	*p*
FAST	9.90 ± 6.19	17.44 ± 7.02	30.64 ± 8.70		29.46	<0.001
SOFAS	77.40 ± 6.90	69.22 ± 9.51	55.82 ± 10.75		19.85	<0.001
SDS	6.10 ± 3.63	10.39 ± 4.17	14.22 ± 7.67		48.91	<0.001

## Discussion

The Finnish version of FAST showed similar excellent psychometric properties as the original version regarding internal consistency and test-retest reliability. Furthermore, the correlation with well-known previous scales assessing functioning, namely the SOFAS and the SDS, was high.

In addition to Spanish and English, the FAST has been translated into Italian (Moro et al. 2012, Barbato et al. 2013), Portuguese (Cacilhas et al. 2009), and Turkish (Aydemir and Uykur 2012) and now also Finnish. The psychometric properties of the Finnish version of FAST showed high internal consistency, in accord with earlier studies. Furthermore, the reliability of the scale, as evaluated by two different clinicians blind to each other’s findings, was excellent (correlation coefficient 0.896). FAST includes the following six areas of functioning: autonomy, occupational functioning, cognitive functioning, financial issues, interpersonal relationships, and leisure time. The reliability for domains regarding autonomy, occupational functioning, and cognitive functioning was excellent, whereas for domains regarding financial issues, interpersonal relationships, and leisure time, the reliability was good. These domains are potentially more difficult to assess over a short period. We also compared FAST with the commonly used and well-known scales of SOFAS and SDS. The correlations between FAST and SOFAS and between FAST and SDS were good. In addition, the correlation between FAST occupational functioning and SDS work domain was good. Finally, we investigated the validity of FAST according to employment status and experienced work ability. The scores of employed subjects were significantly lower than the scores of subjects with sick leave or disability pension. Also, subjective ability to work correlated well with FAST scores. Thus, the results confirm previous findings that higher scores in FAST are associated with poorer functioning.

This study has some limitations. We did not have healthy controls to analyse the scale’s capacity to discriminate between patients and controls. However, previous studies have examined this and have reported the optimal cutoff on the FAST total score for discriminating patients from controls to be 11 (Rosa et al. 2007, Moro et al. 2012) or 15 (Barbato et al. 2013). The sample size of the study was also modest. However, our results were statistically highly significant and similar to those of earlier studies. Furthermore, we did not investigate sensitivity to change, which is a critical feature of a scale in outcome studies, and it should be investigated in the future. However, in other studies, the original FAST has demonstrated sufficient sensitivity to change (Rosa et al. 2011; Torrent et al. 2013). Finally, the translation was conducted by the clinical research group in collaboration with researchers of the Barcelona Bipolar Disorders program. Issues of cross-cultural adaptation (see Epstein et al. 2015) were explicitly discussed between the two groups, and back-translation was undertaken, but no focus group or expert committee was available.

## Conclusions

The psychometric validity and reliability of FAST in the Finnish sample of patients with bipolar disorder types I and II were good. The Finnish version of FAST is suitable for assessing functional impairment in bipolar disorder in both research and clinical practice. The study adds to the accumulating international literature documenting the validity of translated versions of the FAST in evaluation of impairment of patients with bipolar disorder.
